# Integrative Data Mining Highlights Candidate Genes for Monogenic Myopathies

**DOI:** 10.1371/journal.pone.0110888

**Published:** 2014-10-29

**Authors:** Osorio Abath Neto, Olivier Tassy, Valérie Biancalana, Edmar Zanoteli, Olivier Pourquié, Jocelyn Laporte

**Affiliations:** 1 Dept. of Translational Medicine and Neurogenetics, IGBMC, INSERM U964, CNRS UMR7104, University of Strasbourg, Collège de France, Illkirch, Strasbourg, France; 2 Departamento de Neurologia, Faculdade de Medicina de São Paulo (FMUSP), São Paulo, Brazil; 3 Dept. of Development & Stem Cells, IGBMC, INSERM U964, CNRS UMR7104, University of Strasbourg, Collège de France, Illkirch, Strasbourg, France; 4 Faculté de Médecine, Laboratoire de Diagnostic Génétique, Nouvel Hopital Civil, Strasbourg, France; University of Jaén, Spain

## Abstract

Inherited myopathies are a heterogeneous group of disabling disorders with still barely understood pathological mechanisms. Around 40% of afflicted patients remain without a molecular diagnosis after exclusion of known genes. The advent of high-throughput sequencing has opened avenues to the discovery of new implicated genes, but a working list of prioritized candidate genes is necessary to deal with the complexity of analyzing large-scale sequencing data. Here we used an integrative data mining strategy to analyze the genetic network linked to myopathies, derive specific signatures for inherited myopathy and related disorders, and identify and rank candidate genes for these groups. Training sets of genes were selected after literature review and used in Manteia, a public web-based data mining system, to extract disease group signatures in the form of enriched descriptor terms, which include functional annotation, human and mouse phenotypes, as well as biological pathways and protein interactions. These specific signatures were then used as an input to mine and rank candidate genes, followed by filtration against skeletal muscle expression and association with known diseases. Signatures and identified candidate genes highlight both potential common pathological mechanisms and allelic disease groups. Recent discoveries of gene associations to diseases, like *B3GALNT2, GMPPB* and *B3GNT1* to congenital muscular dystrophies, were prioritized in the ranked lists, suggesting *a posteriori* validation of our approach and predictions. We show an example of how the ranked lists can be used to help analyze high-throughput sequencing data to identify candidate genes, and highlight the best candidate genes matching genomic regions linked to myopathies without known causative genes. This strategy can be automatized to generate fresh candidate gene lists, which help cope with database annotation updates as new knowledge is incorporated.

## Background

A large number of disorders affecting skeletal muscle have a genetic basis, with multiple modes of inheritance. They are classified based on phenotype and histopathological features into several groups, which include muscular dystrophies, congenital myopathies and myotonic syndromes, among others (Table 1) [1]. Muscular dystrophies and congenital muscular dystrophies, for example, are characterized by dystrophic changes on muscle biopsy, as opposed to congenital myopathies, which have non-dystrophic peculiar histopathologic findings [2]–[5]. Despite being rare, most inherited myopathies impose a heavy burden on the life of affected persons, and have a strong impact on the health care system. The identification of the causative gene and mutations is often a pre-requisite for genetic counseling and potentially prenatal diagnosis, improved disease care, and access to more specific therapies or inclusion in clinical trials. A lot of advances have been made in the last few decades on the molecular bases of inherited myopathies, which included the discovery of about 130 genes associated with different disorders [1]. Still, it is estimated that around 40% of patients afflicted with myopathies remain without a molecular diagnosis, supporting the implication of additional genes [6], [7]. Further identification of these genes is the focus of a tremendous research effort at present, and will help understand pathological mechanisms and defining novel drug targets.

**Table 1:** Breakdown of disease groups and known associated genes.

Disease group	Main diseases	Associated genes
Muscular dystrophies	Duchenne and Becker musculardystrophies, Emery-Dreifussmuscular dystrophy, Limb-girdle muscular dystrophies	*ANO5, CAPN3, CAV3, DAG1, DES, DMD, DNAJB6, DPM3, DUX4, DYSF, EMD, FHL1, FKRP, FKTN, LMNA, MYOT, PABPN1, PLEC, POMGNT1, POMT1, POMT2, PTRF, SGCA, SGCB, SGCD, SGCG, SYNE1, SYNE2, TCAP, TMEM43, TNPO3, TRAPPC11, TRIM32, TTN*
Congenital musculardystrophies	Merosin-deficient CMD,Dystroglycanopathies, Ulrich andBethlem myopathies	*CHKB, COL6A1, COL6A2, COL6A3, DNM2, DPM2, FHL1, FKRP, FKTN, GTDC2, ISPD, ITGA7, LAMA2, LARGE, LMNA, POMGNT1, POMT1, POMT2, SEPN1, TCAP*
Congenital myopathies	Centronuclear myopathy, Nemalinemyopathy, Central core disease	*ACTA1, BIN1, CCDC78, CFL2, CNTN1, DNM2, KBTBD13, KLHL40, MEGF10, MTM1, MTMR14, MYH2, MYH7, NEB, RYR1, SEPN1, STIM1, TNNT1, TPM2, TPM3, TRIM32, TTN*
Metabolic myopathies	Glycogen storage diseases (Pompe,McArdle), Lipid storage diseases(CPTII deficiency)	*ACADVL, AGL, CPT2, ENO3, GAA, GBE1, GYG1, GYS1, LDHA, LPIN1, PFKM, PGAM2, PGK1, PGM1, PHKA1, PNPLA2, PYGM, SLC22A5, SLC25A20*
Congenital myastenic syndromes	Acetylcholine receptor deficiency,Choline acetyl transferase deficiency,Escobar syndrome	*AGRN, CHAT, CHRNA1, CHRNB1, CHRND, CHRNE, CHRNG, COLQ, DOK7, DPAGT1, GFPT1, LAMB2, MUSK, RAPSN, SCN4A*
Myotonic syndromes	Myotonic dystrophy type 1 (Steinertdisease), Schwartz-Jampel disease	*ATP2A1, CAV3, CNBP, DMPK, HSPG2*
Ion channel muscle diseases	Myotonia congenita, Hyperkalemicperiodic paralysis, Paramyotoniacongenita	*CACNA1S, CLCN1, SCN4A*
Vacuolar myopathies	Myopathy with excessive autophagia,Danon disease, Inclusion bodymyopathy with Paget disease of bone and frontotemporal dementia	*EPG5, GNE, LAMP2, VCP, VMA21*
Myofibrillar myopathies	Alpha-B-crystallin related myofibrillarmyopathy, Desmin related myofibrillarmyopathy	*BAG3, CRYAB, DES, FLNC, LDB3, MYOT, SEPN1*

Next-generation sequencing (NGS) is a relatively new technology that enables massive parallel sequencing of a huge number of bases. It has revolutionized molecular diagnosis and genetic research, as it represents a cost-effective way of testing several genes at once in disorders with genetic heterogeneity, such as myopathies [8]–[10]. Moreover, exome sequencing (ES) or genome sequencing (GS) aid in the discovery of new genes associated with various diseases [11], [12]. There has recently been a surge in publications that use NGS to discover new genes associated with diseases, including myopathies [13]–[20].

The biggest challenge of NGS is to cope with the complexity of analyzing the massive amount of variants generated by the approach. Indeed, comparing two unrelated individuals may lead to about 3 million matching variants in their genomes or about 20,000 in their exomes, but only one of these variants can cause a monogenic disease. The resolution of this issue demands good filtering pipelines to exclude common or meaningless variants, based on the biochemical function of genome location as studied through the ENCODE project [21], and on relationships between human variations and phenotype as in ClinVar and in locus specific databases [22], [23]. In addition, ranking systems can help prioritize validation of the most promising variants. It makes sense to focus on genes presumably implicated in the disease process via functional, structural or phenotypical links with known genes. One of the approaches to collect and compare these data is via *in silico* analysis using a multitude of open-access knowledge information sources. This approach has been recently done successfully for some disorders but not yet for myopathies [24], [25]. Lists of candidate genes thus generated can be ranked and used to prioritize variants resulting from NGS analysis.

Here, we propose ranked lists of candidate genes for individualized groups of inherited myopathies and related diseases that were obtained via data mining of online information databases. These lists can be coupled to NGS analyses pipelines to help filter and prioritize variants aiming at the discovery of novel genes. We also put forward a number of genetic and functional insights taken from the generation of signatures for such disease groups to suggest common pathological pathways between them that can be subject of further scrutiny.

## Methods

### Classification of myopathy genes into 9 overlapping disease groups

The disease groups and associated known genes were based on a modified version of the Gene Table of Neuromuscular Disorders (GTNMD) [26]. We selected the following disease groups, which are primarily related to skeletal muscle pathology: Muscular Dystrophies, Congenital Muscular Dystrophies, Congenital Myopathies, Myotonic Syndromes, Ion Channel Muscle Diseases, Metabolic Myopathies, and Congenital Myasthenic Syndromes. To cope with an ill-defined classification of “Other Myopathies” in the GTNMD, we decided to cluster genes from this group into two new disease groups, Myofibrillar Myopathies and Vacuolar Myopathies. A literature search was performed to find recently published genes not yet listed in the Gene Table version that was used in our present study, which resulted in the addition of the following genes: *VMA21* [16] and *EPG5* [14] to the Vacuolar Myopathies group; *TRAPPC11* [13] and *TNPO3* [19], [27] to the Muscular Dystrophies group; and *STIM1* [20], *CCDC78* [15] and *KLHL40* [17] to the Congenital Myopathies group.

The disease groups have some degree of overlap due to phenotypic heterogeneity of certain genes. For example, *SEPN1* is implicated in multi-minicore disease (a congenital myopathy), and in rigid-spine muscular dystrophy (a congenital muscular dystrophy); *CAV3* both causes limb-girdle muscular dystrophy 1C (a muscular dystrophy), and rippling muscle disease (a myotonic syndrome). The largest overlap is found between Muscular Dystrophies and Congenital Muscular Dystrophies, with 8 genes out of 34 muscular dystrophy-associated genes also found among 20 congenital muscular dystrophy-associated genes.

GTNMD's disease group "Distal Myopathies" was not included as a separate class in this work due to the lack of a gene uniquely associated with it - all genes were also found in other disease groups. Non-myopathy disease groups, such as ataxias, neuropathies, and motor neuron diseases, were also not included, as well as genes that, although listed in the GTNMD under included disease groups, do not lead to a skeletal muscle phenotype. This was the case of *MYBPC3*, implicated in cardiomyopathies, removed from the Congenital Myopathies group; *PRKAG2*, which causes a glycogen storage disease of the heart, not included in the Metabolic Myopathies group; and genes excluded from the Ion Channel Muscle Diseases group because they lead to various ataxia and cardiac arrhythmia syndromes, while not resulting in periodic muscle paralysis. The full list of genes and disease groups used in this work can be found in Table 1.

### Data-mining from online databases to address complex biological questions

We used the data mining system Manteia [28], a public resource available online (manteia.igbmc.fr) that retrieves and combines data from freely available online data sources such as Ensembl, Reactome, OMIM, NCBI, Human Phenotype Ontology (HPO), Gene Ontology (GO), Mouse Genome Informatics, and InterPro. Manteia makes it possible to address complex biological questions by running several queries at the same time to mine and statistically analyze gene sets to highlight their annotation specificities compared to the rest of the genome. This study was conducted with Manteia version 2 with data downloaded in June 2013 from the different databases used in the system.

Using Manteia’s orthology module, we analyzed human gene sets and their mouse orthologs to find an enrichment on statistically significant terms within several annotation categories, including Gene Ontology (GO), Human Phenotype Ontology (HPO), Mammalian Phenotype Ontology (MPO), pathways (Reactome), protein motifs (Interpro) and interacting complexes (Reactome). Gene length was not taken into account as there is no clear enrichment of large genes mutated in myopathies; while some large genes are indeed implicated (*TTN*, *NEB*), smaller genes were found to accumulate mutations along their sequence (e.g. *ACTA1*). Manteia calculates the enrichment of each term in the gene set compared to all genes in the genome, and sorts the terms according to individual statistical significances.

The list of specific terms for each data set can then be used to screen the genome looking for genes that have similar properties. This is achieved using a query builder, which outputs a list of candidate genes ranked according to their similarity with the data set annotation signature and the weight given for each term.

### Extraction of specific signatures for each disease group based on known genes

Statistical analysis for human genes in each disease group was individually performed for Gene Ontology (GO) terms, Human Phenotype Ontology (HPO) terms, pathways, complexes and protein motifs. Mouse orthologs were additionally used to get statistical breakdowns of Mammalian Phenotype Ontology (MPO) terms.

Signatures were represented by a weighted combination of GO terms, HPO terms, MPO terms, and what were collectively called "Interactions Annotation" (IA) terms - pathways and protein complexes descriptors from Reactome and descriptors of protein motifs from InterPro. For each disease group, terms were chosen from the various domains in order to obtain a signature of the disease group. We used the following criteria to select GO terms, HPO terms and MPO terms for each group: 1) significance *p*-value less than 0.05 (corrected using the Benjamini-Hochberg false discovery rate (FDR) procedure); 2) occurrence in the disease group gene set greater than 1; 3) occurrence in the genome <800; 4) GO level (or HPO level or MPO level) >2.

The FDR-BH correction of the *p*-value was chosen because it reduced the large size of the list of resulting terms while not being as stringent as the Bonferroni correction. Terms with only one occurrence in the gene set were deemed not representative of the set. Criteria 3 and 4 enrich for specificity and are closely associated owing to less specific terms (higher ontology level) being associated with a large number of genes; such general terms would not only be unproductive in compounding a signature for a disease group, but also could degrade the performance of a complex query.

For IA terms, the restriction on the occurrence of only one gene in the set was dropped with the aim of improving the scores of new genes related to protein function linked to single known myopathy gene. Indeed, a large proportion of significant terms have only one occurrence in any given disease group gene set. Finally, criteria 4 does not apply to Interpro and Reactome data, which are not structured in defined hierarchies as gene and phenotype ontologies.

### Ranking formula based on weighted scores of signature terms

**Figure 1:**
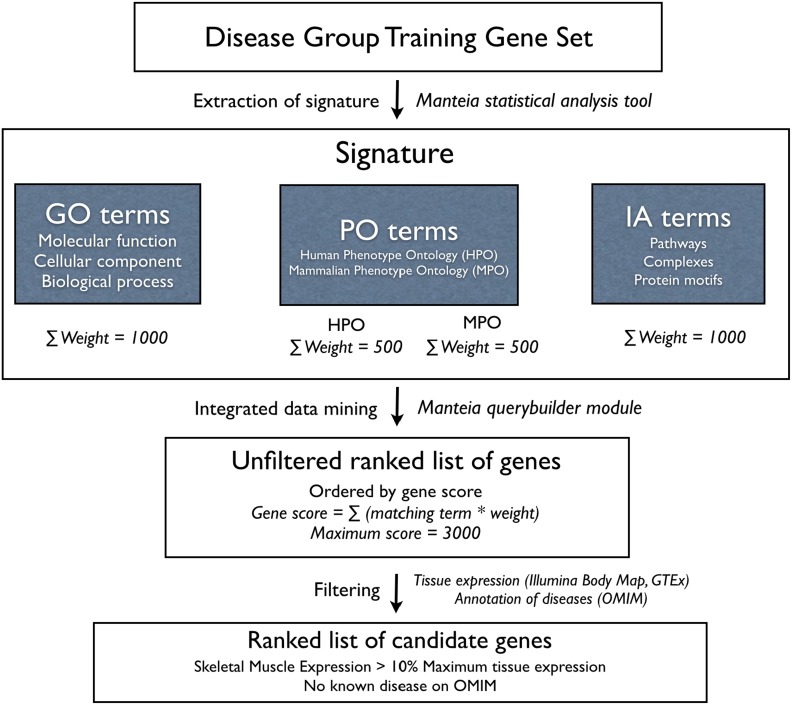
Integrated data mining workflow. A signature of a disease group, composed of weighted terms, is generated from statistical analyses of genes already implicated in diseases of the group. Terms come from the three main annotation groups, GO (Gene Ontology), PO (Phenotype Ontology, an aggregate of Human Phenotype Ontology and Mammalian Phenotype Ontology) and IA (Interactions Annotation), are mined using Manteia and receive weights proportional to the their enrichment in the set of genes implicated in the disease group, as compared to the set of all genes in the human genome. Weights are attributed to terms so that annotation groups contribute equally to the composition of the signature. The signature of the disease group is then used to mine the genome for additional genes. Every gene in the genome receives a score equal to the sum of weights of terms that describe the gene if they match terms that define the disease group signature, for a maximum possible score of 3000. Further filtering steps mark genes that have low relative skeletal muscle expression or are annotated with known diseases.

After experimenting with different signature definitions, we decided to define a signature as having an equal contribution of GO terms, phenotype terms (HPO and MPO terms) and IA terms (Figure 1), so as not to *a priori* give more importance to any term set. A signature with a stronger component of phenotype terms, for example, yields a list of purported candidates strongly biased to genes implicated in known diseases or for which mouse models have been extensively phenotyped. Likewise, if GO terms are the main component of the signature, genes with functional links are preferentially ranked. Finally, IA terms boost the interactome of the known genes to the top of the ranked lists.

Thus, in our approach, for each disease group, the weight of individual terms was calculated so that the added weights of all GO terms was the same as the total PO score (added HPO terms weights combined with added MPO terms weights) and as the added weights of all IA terms, which was arbitrarily set as 1000.

In the GO domain, we defined strata of term weights with percentile cutoffs so that terms with a higher significance would respond for a larger share of the total GO score. The top 20% of terms (p80) contributed to 40% of the total GO score, a middle tier comprising 40% of terms (p40-p80) contributed to an additional 40% of the total GO score, and the lower 40% of terms (p40) provided 20% of the total GO score.

A similar approach was used to calculate individual weights for HPO and MPO terms, with the exception that, as the total PO score reflects the combination of equal shares of HPO and MPO terms, the maximum score of either HPO terms or MPO terms was set at 500.

The weight of each IA term, on the other hand, was the same no matter its position on the corresponding list, and was simply calculated as 1000 divided by the number of significant IA terms for each disease group. This approach helped mine all genes that interacted with any single gene in the training set, provided pathways and interactions were statistically significant.

While the choice of percentile cutoffs that define the strata of weights was arbitrary, we observed that the modification of the cutoffs did not result in substantially different ranked candidate lists for each disease group, as long as the signature definition is the same. All terms for every domain in each disease group, with their corresponding calculated weights, can be found in Table S1.

### Generation of ranked lists of candidate genes for each disease group

Manteia's query builder feature was used to filter genes in the human genome that matched the signature defined for each disease group. Queries combining terms that constitute the signature were run to obtain a list of genes ranked by a score represented by the sum of all matched term weights (Figure 1). More specifically, a gene score results as the sum of a GO score (sum of weights of the disease group's signature GO terms that match the gene's GO terms), a PO score (sum of weights of matching HPO and MPO terms), and an IA score (sum of weights of matching IA terms). The maximum total score a gene can receive is thus 3000 (1000 for GO score+1000 for PO score+1000 for IA score). To deal with MPO terms applied to murine orthologs, the predicted best human orthologs were selected using Manteia's ORTHO function after the ranking process. The ranked lists for each of the 9 disease groups, including annotation detailed in the next subsection, can be found in Table S2.

### Additional filtering of ranked candidate genes using expression data and association with human diseases

The ranked lists for each disease group include the known genes of the group, which were used to create the signatures, genes known to be associated with myopathies but implicated in other disease groups, and genes that are not listed in any disease group and thus represent potentially good candidate genes for myopathies. Among those, additional filtering was performed using tissue expression databases. Data from Illumina Body Map E-MAT513, established from mRNA-Seq of 16 human tissues, was downloaded for every gene, and genes with no expression in skeletal muscle or with an expression in skeletal muscle that was less than a cutoff of 10% of the maximum expression found in any other tissue were excluded. The rationale behind this filtering is that if a gene is expressed in a tissue other than skeletal muscle at a much higher level, one expects such a gene to be implicated in disorders primarily involving that tissue. The 10% cutoff was empirically determined due to the fact that all genes already implicated in myopathies have skeletal muscle expression levels above this cutoff. To deal with missing expression data and eventual heterogeneity in Illumina Body Map's expression database, genes ruled out by the 10% threshold and candidate genes within the 100 first positions in the rankings for each disease group were double checked using expression data from the Genotype-Tissue Expression Project (GTEx) [29].

For the Congenital Myasthenic Syndromes disease group, which includes diseases primarily related to neuromuscular junction protein defects, but also some peripheral nerve terminal protein defects, we decided to disregard the muscle expression filtering due to the fact that the implicated genes *AGRN*, *CHAT*, and *CHRNE* do not have significant skeletal muscle expression (they are instead expressed in the nerve terminal).

The lists of candidate genes after skeletal muscle expression filtration was further annotated with Online Mendelian Inheritance in Men (OMIM) data on existing human phenotype in the form of well-characterized diseases or syndromes, in order to easily identify genes biased by phenotype, such as *SMN1,* which results in a phenotype very similar to many myopathies, characterized by flaccid proximal limb weakness, but which gives rise instead to a motor neuron disease; or biased by interactions, as occurs to a number of carbohydrate metabolism genes that share common pathways to metabolic myopathies but cause instead inborn errors of metabolism without a muscle phenotype.

## Results

### Myopathy groups are clustered by gene ontology and protein function

To identify novel candidate genes for myopathies, we established an integrated data mining approach aiming first to extract specific signatures for disease groups encompassing previously implicated genes, and then to use these signatures to search for additional matching genes in the human genome. As detailed in Figure 1 and the methods section, this approach consists of a weighted ranking of three main sets of data: gene ontology, human and mouse phenotypes ontologies, and “interactions annotation” incorporating pathways and protein motifs and complexes.

**Figure 2:**
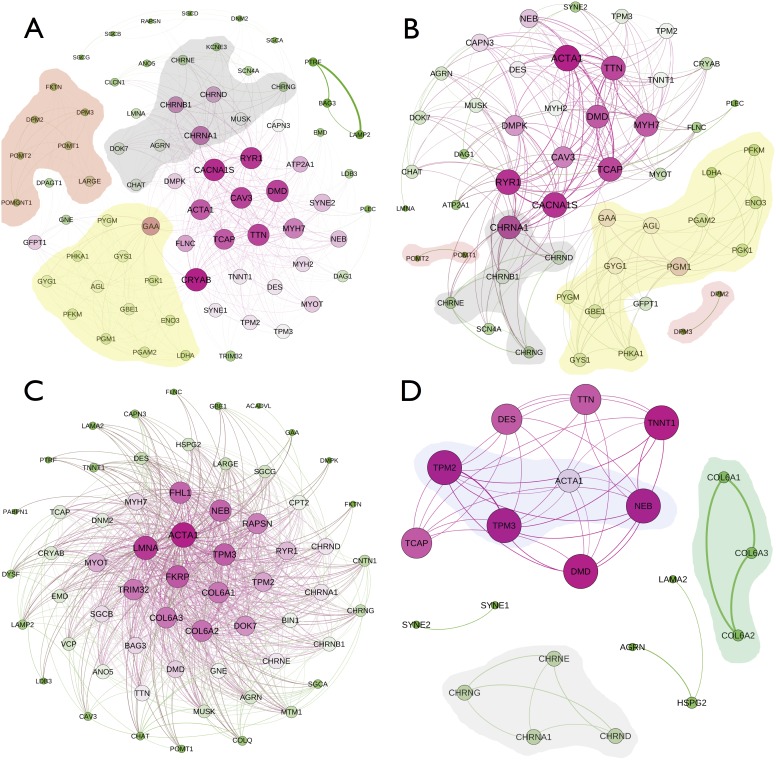
Graph representation of relationships of known genes. All known genes for the different disease groups were concurrently analyzed for matching terms in different ontologies. Nodes represent genes, and edges between two given nodes are depicted when the number of terms shared by the two connected genes is greater than a certain threshold. Edge width is proportional to the number of terms shared between two genes, and node size and color in a scale from green (lowest) to red (highest) is proportional to the number of associations of a gene in the graph. Closely related genes appear clustered together, and hubs in the graph appear centrally located. A: graph for combined terms from Gene Ontology (GO), Human Phenotype Ontology (HPO) and Interactions Annotation (IA), with a threshold of 30 matching terms. The cluster with a yellow background includes genes implicated in metabolic myopathies, the one with a red background groups congenital muscular dystrophy genes, and the cluster with a gray background represents genes associated with congenital myasthenic syndromes. B: graph for HPO terms with a threshold of 20 matching terms. C: graph for GO terms, with a threshold of 10 matching terms. Background colors correspond to clusters represented in A. D: IA terms with a threshold of 5 matching terms. The gray background highlights a cluster with gene that code subunits of cholinergic receptors, implicated in congenital myasthenic syndromes, the green one groups components of collagen VI, and the cluster with a blue background links elements of the contractile apparatus.

To test this approach and better visualize signature composition analysis, we first analyzed a training set that consisted in all myopathy-associated genes using the data mining system Manteia [28]. Figure 2 shows graphs with relationships between all known genes of the nine chosen disease groups. In particular, the combination of GO, PO and IA terms aggregate most genes that are part of the same myopathy group for metabolic myopathies, the congenital myasthenic syndromes, and the glycosylation components of congenital muscular dystrophies (Figure 2A). Of note, the gene *GFPT1*, which causes a congenital myasthenic syndrome with tubular aggregates, has mainly relationships with genes in the metabolic myopathy cluster, presumably because it codes for an enzyme in the metabolism of glycoproteins. Another large cluster in the graph encompasses the main genes implicated in muscular dystrophies and congenital or myofibrillar myopathies, without subdivision, suggesting a strong overlap in the function of the related genes and potentially in the pathogenesis. This approach can thus retrieve several phenotypic and pathologic clusters. However, applying only the human phenotype ontology analysis generates a single large, highly connected graph (Figure 2B), even when the threshold for representing an edge in the graph - number of matching HPO term between two genes - is increased or decreased, or when HPO term hierarchy is taken into account. This means that genes implicated in myopathies share a common hierarchy of phenotype ontology terms, e.g. with most genes annotated with muscle weakness or abnormal muscle physiology related terms. While they do not help separate genes into disease groups, HPO terms are important to help emerge genes with phenotype annotation associated to skeletal muscle. GO terms and IA terms, on the other hand, are responsible for the final clustering. Different myopathy groups appear using only GO terms (Figure 2C), while IA terms, even considering a lower threshold of 5 terms shared between genes, create smaller clusters of genes that interact closely by sharing the same pathways, interactions complexes or motifs, such as constituents of collagen VI, genes responsible for the assembly of nicotinic cholinergic receptors, or conglomerated proteins involved with the sarcomere (Figure 2D). Only the combination of the different GO, PO and IA terms reaches the most precise clustering.

### Characterization of disease groups via biological processes annotation

We next aimed to extract specific signatures for each disease group, classified based on the Gene Table of Neuromuscular Disorders [1]. Statistical analysis of known genes was conducted for each disease group.

GO terms include three types of ontologies: cellular components indicate the localization of gene products; molecular function refers to the normal roles of genes at the molecular level; and biological processes describe the higher-order roles of genes from a biological perspective. Four main general skeletal muscle-related biological processes were extrapolated from the hierarchy of GO terms: muscle contraction, calcium homeostasis, muscle development, and muscle intracellular organization (Table 2). Analysis of the breakdown of biological process-related GO terms that make up the signatures of different disease groups reveals differences in the implicated skeletal muscle processes and hints on other important biological processes that do not primarily involve the skeletal muscle.

**Table 2:** Composition of biological processes GO terms that make up the signature of each disease group.

DiseaseGroup	Musclecontraction	Calciumhomeostasis	Muscledevelopment	Muscleintracellularorganization	Otherterms	Total	Other term categories
Congenitalmyopathy	7	10	12	3	13	45	Cardiac development,catabolism of nucleotides
Musculardystrophy	9	1	14	23	30	77	Glycosylation, cardiac development,cardiac contraction
Congenital musculardystrophy	0	0	2	0	20	22	Cardiac development,glycosylation
Metabolic myopathy	1	1	0	0	73	75	Glycogen metabolism
Congenital myasthenicsyndrome	2	7	18	2	64	93	Neuromuscular junction,synapses
Myotonic myopathy	7	21	11	2	9	50	Heart contraction,circulation
Ion channelmuscle disease	1	4	0	0	2	7	Ion transport

Vacuolar myopathies and myofibrillar myopathies did not receive in their signature GO terms associated with biological processes, because the training set genes for these groups were annotated with heterogeneous terms that did not attain statistical significance. Metabolic myopathies and congenital myasthenic syndromes inferred biological processes were, as expected, not primarily muscle related, but had mostly to do with glycogen metabolism and neuromuscular junction, respectively. Congenital muscular dystrophies, while having two GO terms associated with muscle development, were also primarily annotated with non muscle-specific biological processes, especially protein glycosylation. Myotonic myopathies and ion channel muscle diseases take the larger contribution from calcium homeostasis-related terms. Muscular dystrophies mainly involve muscle intracellular organization terms, but also receive some contribution from muscle development and muscle contraction terms. Other important biological processes for muscular dystrophies are associated with heart muscle contraction and development. Finally, for congenital myopathies, muscle development and calcium homeostasis seem to be the most significant processes, but muscle contraction-related terms also play a role, as well as processes not specific for skeletal muscle, such as catabolism of nucleotides - these appear enriched due to the association of *DNM2* to the catabolism of GTP, as well as *MYH7* and *TPM2* to the catabolism of ATP.

### Training set genes appear at the top of the ranked lists of the disease groups

We used the signature specific to each disease group to screen the whole set of human genes and identify candidate genes for myopathies. The breakdown of the gene score for these training set genes shows that a similar contribution of the different term domains can be consistently found throughout the various disease groups. GO score, PO score and IA score respond for approximately 30 to 45%, 40 to 55%, and 10 to 15% of the gene score, respectively. Table 3 shows the ranked lists of known genes for three disease groups (Congenital Myopathies, Muscular Dystrophies and Metabolic Myopathies), along with each gene score and breakdown of partial scores. Table S3 shows similar additional data for all disease groups.

**Table 3:** Score breakdown for training sets of congenital myopathies, muscular dystrophies and metabolic myopathies disease groups.

Muscular dystrophies											
Rank	Gene	Gene Score	GO score	%GO	HPO score	%HPO	MPO score	%MPO	%PO	IA score	%IA
1	*DMD*	1115	352	31.57	180	16.14	375	33.63	49.78	208	18.65
2	*TTN*	1064	520	48.87	224	21.05	208	19.55	40.60	112	10.53
3	*LMNA*	997	208	20.86	361	36.21	284	28.49	64.69	144	14.44
4	*TCAP*	915	508	55.52	209	22.84	86	9.40	32.24	112	12.24
5	*DES*	814	252	30.96	195	23.96	223	27.40	51.35	144	17.69
6	*CAV3*	777	384	49.42	181	23.29	196	25.23	48.52	16	2.06
11	*SYNE2*	599	340	56.76	83	13.86	16	2.67	16.53	160	26.71
13	*SYNE1*	577	236	40.90	87	15.08	110	19.06	34.14	144	24.96
17	*FKRP*	560	48	8.57	333	59.46	163	29.11	88.57	16	2.86
18	*DAG1*	553	288	52.08	0	0.00	233	42.13	42.13	32	5.79
19	*PLEC*	552	140	25.36	65	11.78	171	30.98	42.75	176	31.88
20	*CAPN3*	539	176	32.65	123	22.82	176	32.65	55.47	64	11.87
22	*TRIM32*	518	92	17.76	299	57.72	95	18.34	76.06	32	6.18
23	*SGCB*	518	112	21.62	245	47.30	145	27.99	75.29	16	3.09
24	*SGCA*	503	136	27.04	163	32.41	156	31.01	63.42	48	9.54
25	*EMD*	487	196	40.25	180	36.96	79	16.22	53.18	32	6.57
28	*SGCG*	471	112	23.78	136	28.87	207	43.95	72.82	16	3.40
32	*MYOT*	446	176	39.46	270	60.54	0	0.00	60.54	0	0.00
36	*DYSF*	416	36	8.65	155	37.26	161	38.70	75.96	64	15.38
80	*POMT1*	278	96	34.53	116	41.73	18	6.47	48.20	48	17.27
89	*FHL1*	270	32	11.85	238	88.15	0	0.00	88.15	0	0.00
92	*SGCD*	266	112	42.11	0	0.00	138	51.88	51.88	16	6.02
94	*POMGNT1*	265	76	28.68	92	34.72	81	30.57	65.28	16	6.04
114	*ANO5*	243	0	0.00	227	93.42	0	0.00	93.42	16	6.58
130	*FKTN*	226	84	37.17	112	49.56	14	6.19	55.75	16	7.08
193	*PABPN1*	181	40	22.10	77	42.54	0	0.00	42.54	64	35.36
262	*POMT2*	154	88	57.14	0	0.00	18	11.69	11.69	48	31.17
282	*PTRF*	149	16	10.74	124	83.22	8	5.37	88.59	0	0.00
443	*DPM3*	112	80	71.43	0	0.00	0	0.00	0.00	32	28.57
	Average	502.24	170.21	32.68	154.31	34.51	115.90	20.30	54.81	61.79	12.48
**Congenital myopathies**											
**Rank**	**Gene**	**Gene Score**	**GO score**	**%GO**	**HPO score**	**%HPO**	**MPO score**	**%MPO**	**%PO**	**IA score**	**%IA**
1	*TTN*	1309	641	48.97	206	15.74	315	24.06	39.80	147	11.23
2	*ACTA1*	1291	555	42.99	441	34.16	169	13.09	47.25	126	9.76
3	*RYR1*	1162	349	30.03	292	25.13	332	28.57	53.70	189	16.27
4	*NEB*	1146	364	31.76	370	32.29	160	13.96	46.25	252	21.99
5	*TPM3*	1090	425	38.99	371	34.04	0	0.00	34.04	294	26.97
7	*TPM2*	998	393	39.38	311	31.16	0	0.00	31.16	294	29.46
11	*MYH7*	917	635	69.25	198	21.59	0	0.00	21.59	84	9.16
16	*TRIM32*	758	234	30.87	279	36.81	203	26.78	63.59	42	5.54
22	*MTM1*	702	53	7.55	211	30.06	354	50.43	80.48	84	11.97
27	*TNNT1*	663	333	50.23	141	21.27	0	0.00	21.27	189	28.51
33	*DNM2*	618	89	14.40	200	32.36	224	36.25	68.61	105	16.99
44	*MYH2*	527	443	84.06	0	0.00	0	0.00	0.00	84	15.94
55	*BIN1*	454	101	22.25	209	46.04	60	13.22	59.25	84	18.50
103	*SEPN1*	333	0	0.00	0	0.00	333	100.00	100.00	0	0.00
209	*CNTN1*	216	34	15.74	141	65.28	20	9.26	74.54	21	9.72
313	*STIM1*	170	122	71.76	33	19.41	15	8.82	28.24	0	0.00
354	*MTMR14*	160	53	33.13	0	0.00	65	40.63	40.63	42	26.25
	Average	736.12	283.76	37.14	200.18	26.20	132.35	21.47	47.67	119.82	15.19
**Metabolic myopathies**											
**Rank**	**Gene**	**Gene Score**	**GO score**	**%GO**	**HPO score**	**%HPO**	**MPO score**	**%MPO**	**%PO**	**IA score**	**%IA**
1	*GAA*	875	410	46.86	136	15.54	309	35.31	50.86	20	2.29
2	*PFKM*	840	225	26.79	162	19.29	353	42.02	61.31	100	11.90
3	*PHKA1*	831	395	47.53	236	28.40	150	18.05	46.45	50	6.02
4	*GBE1*	822	330	40.15	59	7.18	333	40.51	47.69	100	12.17
6	*ACADVL*	747	140	18.74	275	36.81	222	29.72	66.53	110	14.73
7	*GYS1*	746	370	49.60	0	0.00	296	39.68	39.68	80	10.72
8	*PGM1*	709	450	63.47	159	22.43	0	0.00	22.43	100	14.10
12	*AGL*	666	480	72.07	136	20.42	0	0.00	20.42	50	7.51
14	*PYGM*	624	385	61.70	159	25.48	0	0.00	25.48	80	12.82
19	*GYG1*	590	480	81.36	0	0.00	0	0.00	0.00	110	18.64
24	*LDHA*	575	200	34.78	190	33.04	95	16.52	49.57	90	15.65
25	*CPT2*	571	205	35.90	316	55.34	0	0.00	55.34	50	8.76
28	*PGAM2*	551	265	48.09	216	39.20	0	0.00	39.20	70	12.70
37	*PGK1*	477	260	54.51	157	32.91	0	0.00	32.91	60	12.58
47	*ENO3*	441	245	55.56	106	24.04	0	0.00	24.04	90	20.41
77	*PNPLA2*	354	10	2.82	0	0.00	334	94.35	94.35	10	2.82
84	*LPIN1*	344	25	7.27	141	40.99	158	45.93	86.92	20	5.81
87	*SLC25A20*	340	190	55.88	120	35.29	0	0.00	35.29	30	8.82
116	*SLC22A5*	311	60	19.29	123	39.55	108	34.73	74.28	20	6.43
	Average	600.74	269.74	43.28	141.63	25.05	124.11	20.89	45.93	65.26	10.78

Gene score is the sum of GO, HPO, MPO and IA scores. Relative contributions of GO, HPO, MPO and IA scores to the gene score are shown in the columns %GO, %HPO, %MPO and %IA, respectively. Training set genes without database annotation received a gene score of 0 and are not shown.

As expected, genes already known to be mutated in the various disease groups, which were used as the training set to create the mining signatures, appear at the top of the ranked lists of data mining. Considering congenital myopathies, out of the 22 genes chosen as the training set, 19 genes appeared in the data mining, while genes *CCDC78*, *KBTBD13*, and *KLHL40* did not have annotation in the databases used at the time of this work. Thirteen of these genes were ranked within the first 100 genes, a coverage of 13/19 (68.4%). The muscular dystrophy group had 31 out of 34 genes of its training set appearing in the data mining list, and of these 23 were found within the first 100 ranked genes (79.3%). In the metabolic myopathy disease group, all 19 genes of the training set were ranked, and 18/19 were found within the top 100 genes in the rank (94.7%). Outliers among the known genes are mostly poorly annotated genes, and genes with no score are actually not annotated at all (see discussion). Thus, the high ranking of most previously implicated genes supports the signature choice having adequately defined the disease group.

### Proposed candidate genes after filtration

A number of candidate genes sharing disease group signatures with known myopathy genes are barely expressed in skeletal muscle or sometimes mutated in other diseases not affecting skeletal muscle. We thus added filtering steps based on tissue expression and known implication in diseases (see methods for details). Table 4 shows the top 8 ranked genes for each disease group after filtration on skeletal muscle expression and absence of link with diseases in OMIM (Online Mendelian Inheritance in Men, omim.org) database. Table S2 lists the full ranked list of genes for each disease group without filtration, but annotated with skeletal muscle expression and OMIM diseases, and can be linked to NGS filtering pipelines to help prioritization of novel gene discovery, as shown in the discussion. In the following paragraphs, we discuss a few genes found as candidates in some of the disease groups, to illustrate the connections between the integrated data mining results and evidence from the literature.

**Table 4:** Top 8 ranked candidate genes for each disease group.

Muscular dystrophies			
Rank	Gene	Name	Score
33	*ITGB1*	integrin, beta 1 (fibronectin receptor, beta polypeptide, antigen CD29 includesMDF2, MSK12)	437
42	*TMOD1*	tropomodulin 1	391
48	*MYL1*	myosin, light chain 1, alkali; skeletal, fast	368
53	*TNNI1*	troponin I type 1 (skeletal, slow)	356
62	*MYH4*	myosin, heavy chain 4, skeletal muscle	332
67	*UTRN*	utrophin	325
72	*TNNC2*	troponin C type 2 (fast)	304
81	*SRF*	serum response factor (c-fos serum response element-binding transcription factor)	278
**Congenital muscular dystrophies**			
**Rank**	**Gene**	**Name**	**Score**
36	*GCNT4*	glucosaminyl (N-acetyl) transferase 4, core 2	458
44	*GALNT1*	UDP-N-acetyl-alpha-D-galactosamine:polypeptide N-acetylgalactosaminyltransferase 1(GalNAc-T1)	444
47	*ST8SIA2*	ST8 alpha-N-acetyl-neuraminide alpha-2,8-sialyltransferase 2	444
51	*OGT*	O-linked N-acetylglucosamine (GlcNAc) transferase	444
53	*GALNT2*	UDP-N-acetyl-alpha-D-galactosamine:polypeptide N-acetylgalactosaminyltransferase 2(GalNAc-T2)	444
55	*SDF2*	stromal cell-derived factor 2	443
57	*ST8SIA6*	ST8 alpha-N-acetyl-neuraminide alpha-2,8-sialyltransferase 6	442
62	*MGAT1*	mannosyl (alpha-1,3-)-glycoprotein beta-1,2-N-acetylglucosaminyltransferase	426
**Congenital myopathies**			
**Rank**	**Gene**	**Name**	**Score**
17	*MYH4*	myosin, heavy chain 4, skeletal muscle	755
29	*MYL1*	myosin, light chain 1, alkali; skeletal, fast	640
31	*TNNI1*	troponin I type 1 (skeletal, slow)	634
37	*RYR3*	ryanodine receptor 3	587
38	*TMOD1*	tropomodulin 1	570
43	*TNNC2*	troponin C type 2 (fast)	527
51	*MYH1*	myosin, heavy chain 1, skeletal muscle, adult	458
58	*MYL6B*	myosin, light chain 6B, alkali, smooth muscle and non-muscle	438
**Metabolic myopathies**			
**Rank**	**Gene**	**Name**	**Score**
10	*PRKAA2*	protein kinase, AMP-activated, alpha 2 catalytic subunit	672
18	*PPP1R3C*	protein phosphatase 1, regulatory subunit 3C	593
21	*MTOR*	mechanistic target of rapamycin (serine/threonine kinase)	588
26	*PRKAB2*	protein kinase, AMP-activated, beta 2 non-catalytic subunit	560
39	*ACACB*	acetyl-CoA carboxylase beta	470
40	*PHKG1*	phosphorylase kinase, gamma 1 (muscle)	470
44	*PPARGC1A*	peroxisome proliferator-activated receptor gamma, coactivator 1 alpha	459
48	*GSK3A*	glycogen synthase kinase 3 alpha	441
**Congenital myasthenic syndromes**			
**Rank**	**Gene**	**Name**	**Score**
11	*CHRNB4*	cholinergic receptor, nicotinic, beta 4 (neuronal)	817
15	*CHRNA6*	cholinergic receptor, nicotinic, alpha 6 (neuronal)	680
19	*CHRNB3*	cholinergic receptor, nicotinic, beta 3 (neuronal)	636
21	*CACNA2D2*	calcium channel, voltage-dependent, alpha 2/delta subunit 2	628
22	*CHRNA9*	cholinergic receptor, nicotinic, alpha 9 (neuronal)	616
40	*ITGB1*	integrin, beta 1 (fibronectin receptor, beta polypeptide, antigen CD29 includes MDF2, MSK12)	441
52	*CHRNA10*	cholinergic receptor, nicotinic, alpha 10 (neuronal)	413
61	*HTR3B*	5-hydroxytryptamine (serotonin) receptor 3B, ionotropic	399
**Ion channel muscle diseases**			
**Rank**	**Gene**	**Name**	**Score**
12	*SCN3A*	sodium channel, voltage-gated, type III, alpha subunit	1416
13	*CACNB1*	calcium channel, voltage-dependent, beta 1 subunit	1378
40	*RYR3*	ryanodine receptor 3	1019
52	*CACNG1*	calcium channel, voltage-dependent, gamma subunit 1	936
55	*CACNA2D1*	calcium channel, voltage-dependent, alpha 2/delta subunit 1	930
63	*CACNA2D3*	calcium channel, voltage-dependent, alpha 2/delta subunit 3	915
70	*KCNQ5*	potassium voltage-gated channel, KQT-like subfamily, member 5	888
71	*KCNA7*	potassium voltage-gated channel, shaker-related subfamily, member 7	888
**Myotonic syndromes**			
**Rank**	**Gene**	**Name**	**Score**
7	*CASQ1*	calsequestrin 1 (fast-twitch, skeletal muscle)	1079
8	*RYR3*	ryanodine receptor 3	954
15	*JPH1*	junctophilin 1	806
21	*MYL1*	myosin, light chain 1, alkali; skeletal, fast	703
26	*CAMK2D*	calcium/calmodulin-dependent protein kinase II delta	658
32	*SYPL2*	synaptophysin-like 2	611
34	*ITGB1*	integrin, beta 1 (fibronectin receptor, beta polypeptide, antigen CD29 includes MDF2, MSK12)	610
47	*MYH4*	myosin, heavy chain 4, skeletal muscle	559
**Myofibrillar myopathies**			
**Rank**	**Gene**	**Name**	**Score**
27	*MYL1*	myosin, light chain 1, alkali; skeletal, fast	984
33	*MYH4*	myosin, heavy chain 4, skeletal muscle	924
35	*MYL12B*	myosin, light chain 12B, regulatory	921
41	*TNNI1*	troponin I type 1 (skeletal, slow)	909
42	*TNNC2*	troponin C type 2 (fast)	909
50	*PDLIM3*	PDZ and LIM domain 3	862
51	*MYO18B*	myosin XVIIIB	845
52	*PDLIM5*	PDZ and LIM domain 5	844
**Vacuolar myopathies**			
**Rank**	**Gene**	**Name**	**Score**
4	*CD63*	CD63 molecule	1160
8	*AP1G1*	adaptor-related protein complex 1, gamma 1 subunit	1006
18	*VAMP7*	vesicle-associated membrane protein 7	1006
20	*MARCH8*	membrane-associated ring finger (C3HC4) 8, E3 ubiquitin protein ligase	1006
22	*ZNRF1*	zinc and ring finger 1, E3 ubiquitin protein ligase	1006
26	*AP1M1*	adaptor-related protein complex 1, mu 1 subunit	1006
27	*AP1B1*	adaptor-related protein complex 1, beta 1 subunit	1006
40	*ABCC4*	ATP-binding cassette, sub-family C (CFTR/MRP), member 4	817

Candidate genes are not associated with disease (as per annotation in OMIM) and are expressed in skeletal muscle with at least 10% of the maximum expression in any tissue, except for congenital myasthenic syndromes, where there was no expression filtering.

Candidate genes for muscular dystrophies display strong links with muscle development, contraction and intracellular organization, expected subcomponents of skeletal muscle-related biological processes terms from the breakdown of GO terms. *ITGB1* codes for a subunit of ubiquitous fibronectin receptors and has a number of suggested functions in different tissues. In skeletal muscle, it has been proposed as a possible target for myostatin in mice myoblast differentiation [30] and is also critical for the development of neuromuscular junctions [31]. *TMOD1* encodes for tropomodulin, a protein that regulates tropomyosin and F-actin organization. Knockout mice present with age-dependent sarcomere misalignment and sarcoplasmic reticulum morphological defects [32]. *MYL1* is involved with early differentiation of fast muscle cells [33] and *TNNI1* codes for the slow-twitch skeletal muscle isoform of troponin I, which has yet to be associated with human diseases even though the fast-twitch isoform is responsible for a subtype of arthrogryposis and the cardiac isoform causes cardiomyopathy syndromes.

Candidate genes for congenital myopathies have a significant overlap with genes proposed for muscular dystrophies, for example for *TMOD1* and *TNNI1*. Among those with a high rank in congenital myopathies are *RYR3* and *MYH1*. *RYR3* codes for a ryanodine calcium release channel with a low Ca^2+^ sensitivity that has a physiologic role in the excitation-contraction coupling of neonatal skeletal muscles and is up regulated in steroid-associated muscle damage [34], while *MYH1* is one of the adult skeletal muscle isoforms of myosin heavy chain that predominates in 2B myofibers. *RYR3* high ranking is boosted by a strong contribution of calcium homeostasis terms, explaining why *RYR3* received a similarly high score in the Myotonic Syndromes and Ion Channel Muscle Diseases groups, which also have a strong component of calcium homeostasis terms. Also in the group of Ion Channel Muscle Diseases, the gene *CACNB1* encodes both the brain and skeletal muscle isoforms of the calcium channel beta subunit, and its loss in mouse is associated to a phenotype similar to that seen in mice with mutations in the known genes *CACNA1S* or *RYR1* [35].

Within congenital muscular dystrophies, the gene *B3GALNT2,* ranked in the 97th place out of 4841 genes with annotation for this group's signature, was recently found to be associated with hypoglycosylation of alpha-dystroglycan and a congenital muscular dystrophy phenotype in humans [18]. Two other genes, *GMPPB*, ranked in 225th, and *B3GNT1*, ranked in 479th, were also implicated in a form of congenital muscular dystrophy with hypoglycosylation of alpha-dystroglycan and Walker-Warburg disease, respectively [36], [37]. These genes had not been used in the training set of genes for congenital muscular dystrophies, and have since been annotated in OMIM, but their high placement in the ranking list validate the proposed data mining strategy and subsequent filtering steps.

### Candidate genes within genomic regions linked to myopathies

A number of neuromuscular diseases have mapped loci awaiting gene identification [1]. Matching the genomic positions of the top 100 candidate genes of each disease group with such mapped loci reveals some interesting candidates (Table 5).

**Table 5:** Candidate genes within genomic regions linked to myopathies and related diseases.

**Linked** **region**	**Phenotypes and asssociated disease symbols**	**Candidate genes**
1q42	Congenital muscular dystrophy with merosindeficiency - MDC1B	*OBSCN*, *GALNT2*
3p22.2-p21.32	Hyalin body myopathy - HBM	*XIRP1*
3p23-21	Congenital muscle dystrophy with joint hyperlaxity	*XIRP1*
7q21-q22	Malignant hyperthermia susceptibility 3 - MHS3	*CACNA2D1*
17q11.2-q24	Malignant hyperthermia susceptibility 2 - MHS2	*SDF2*, *SYNRG*, *CACNB1*, *CACNG1*
19p13	Muscular dystrophy, autosomal dominant,with rimmed vacuoles - MDRV	*CALR*, *PRKACA*, *AP1M1*

The gene *XIRP1*, matching the locus for hyalin body myopathy and congenital muscular dystrophy with joint hyperlaxity, was originally studied in relation to murine cardiac morphogenesis and later shown to bind skeletal muscle actin in *in vitro* assays [38]. Its product, the Xin protein, is skeletal muscle-specific and has recently been put forward as a potentially useful biomarker of muscle damage, which can be used to monitor disease progression and treatment effects in myopathies [39]. *OBSCN* encodes obscurin, a giant sarcomeric signaling protein similar to titin, which has a suspected role in myofibrillogenesis. It is also involved in dystrophin localization and maintenance of sarcolemma integrity [40], and is proposed here as a candidate for congenital muscular dystrophy with merosin deficiency (MDC1B). An additional candidate gene mapped in the linked region is *GALNT2*, a glycosylating enzyme similar to *B3GALNT2* recently found mutated in another form of congenital muscular dystrophy [18], and also involved in the O-glycosylation of peptides in the Golgi apparatus.

Although not directly analyzed in this work, malignant hyperthermia susceptibility regions encompass *CACNG1* and *CACNA2D1*, which are associated with calcium homeostasis and calcium channels, are highly ranked for Ion Channel Muscle Diseases, and are thus interesting candidates. *CACNA2D1*, specifically, has been suggested at least as a modifier of hyperthermia susceptibility in association to other genes [41]. These genes have been excluded in a limited number of families not linked to *RYR1* mutations [42], results which may be revisited with the advent of NGS data. Additionally, another candidate gene, *CACNB1,* has no associated human disease. However, *CACNG1, CACNB1* and *CACNA2D1* encode for subunits of the DHPR calcium channel, which is in direct contact and regulating RYR1 in skeletal muscle, and one mutation in the channel subunit CACNA1S of DHPR was linked to malignant hyperthermia [43].

The *CALR* and *AP1M1* genes both map to 19q13, the locus associated to autosomal muscular dystrophy with rimmed vacuoles. In a recent work, the product of the *CALR* gene, calreticulin, has been shown to localize in cardiomyocyte mitochondria, and its content increases in mouse models with dilated cardiomyopathy [44]. Strikingly, calreticulin was found to be highly expressed in GNE myopathy, a distal myopathy associated with rimmed vacuoles [45]. Also in distal myopathies with rimmed vacuoles, though not necessarily GNE myopathy, adaptin related-proteins subunits, which are normally not marked in the immunohistochemistry of normal muscle, appear inside or on the rims of vacuoles. The *AP1M1* gene codes for the mu subunit of adaptin related-proteins [46].

## Discussion

In this study, an integrated data mining strategy was used to cluster and rank genes with known or potential importance for skeletal muscle, and to provide candidate genes for myopathies and some related diseases. Results from the clustering and ranking highlight pathological pathways specific for disease groups. The list of candidate genes was further filtered based on expression data and association with other diseases, and the ensuing identification of mutations in high-ranked genes for congenital muscular dystrophies (*B3GALNT2*, *GMPPB* and *B3GNT1*) illustrated the validity of this approach.

### Gene clustering and ranking are dependent on database annotations

Proposed genes in the final ranked list have gene scores with a major contribution of GO and IA terms, and eventual contribution of MPO terms. Thus, they represent genes that have mostly functional links with known myopathy genes (IA terms and GO term ontologies for biological processes and molecular functions), but also some degree of product colocalization in the muscle cell, as expected from matching cellular component-related GO terms. When available, data on altered skeletal muscle function in mouse models also tend to contribute to higher scores for proposed candidate genes.

Database annotation can vary from one gene to another, as it is dependent on the history of research for each gene, including both the date when the gene was discovered and the amount of effort spent for its functional characterization. In addition, animal models are generally phenotyped with a specific organ system in mind.

To illustrate the effects of incomplete annotation, the genes *TRAPPC11* and *TNPO3*, recently implicated in muscular dystrophies, were used as components of this disease group training set, but did not impact the results of the gene ranking due to their poor database annotation. Likewise, they were themselves not captured by the signature used for the Muscular Dystrophies disease group. *TRAPPC11* does not appear in the ranking, as it was annotated with only two GO terms that are not significant for muscular dystrophies ("vesicle-mediated transport" and "Golgi apparatus"), it has no annotation for pathways or phenotypes, and its two protein motifs are unique in the genome. Annotation biases also account for higher placements of better-annotated genes that have some kind of overlap with myopathy genes. Such is the case for motor neuron disease-associated genes, which give rise to human and mouse phenotypes that present some degree of phenotypic overlap with myopathies and tend to share many HPO or MPO terms with myopathy phenotypes. In the ranked list of muscular dystrophies, high scores with a predominant contribution of HPO terms were given to the genes *SMN1*, *SMN2*, *ALS2*, *IGHMBP2* and *AR*. These genes are linked to different types of motor neuron disease, which ultimately manifest with muscle weakness and atrophy.

*In silico* approaches need thus periodic revisits to adjust candidate lists based on association of new genes that impact training sets and discovery of new pathways or interactions that change corresponding database annotation, such as the recently published interactome of skeletal muscle proteins centered on proteins that cause limb-girdle muscular dystrophies [47].

### Possible insights into pathological mechanisms

The integrated data mining identified gene signatures revealing common function within specific myopathy groups or between groups, and highlighting pathological mechanisms.

Known and candidate genes for metabolic myopathies, congenital myasthenic syndromes, myotonic syndromes, and ion channel muscle diseases define distinctive functions for each disease group (Table 2). Highly ranked genes for congenital myasthenic syndromes are associated to function not primarily linked to skeletal muscle but point as expected to the neuromuscular junction. The cellular basis of myotonic myopathies and ion channel muscle diseases consists in the alteration of ion homeostasis. Additional genes contributing to glycogen metabolism were identified as good candidate genes for the metabolic myopathies.

Muscle development, muscle contraction and calcium homeostasis are key pathways linked to congenital myopathies; indeed this myopathy group presents generally at or before birth, and is characterized by histological hallmarks reflecting alteration and aggregation of proteins implicated in muscle contraction (nemaline myopathies) or due to primary defects in the excitation-contraction coupling (core myopathies and potentially the centronuclear myopathies).

Muscular dystrophies mainly involve muscle intracellular organization terms that reflect the structural importance of most proteins already reported mutated. However, other pathways may have been overlooked because the way the first genes were discovered; once the *DMD* gene was found, investigators started seeking mutations on genes from the dystrophin-glycoprotein complex. Of note, based on the terms breakdown and candidate genes identified, muscular dystrophies may have a larger contribution from the contractile apparatus than previously assumed, which would bring this disease group closer to congenital myopathies.

### Allelic diseases

**Figure 3:**
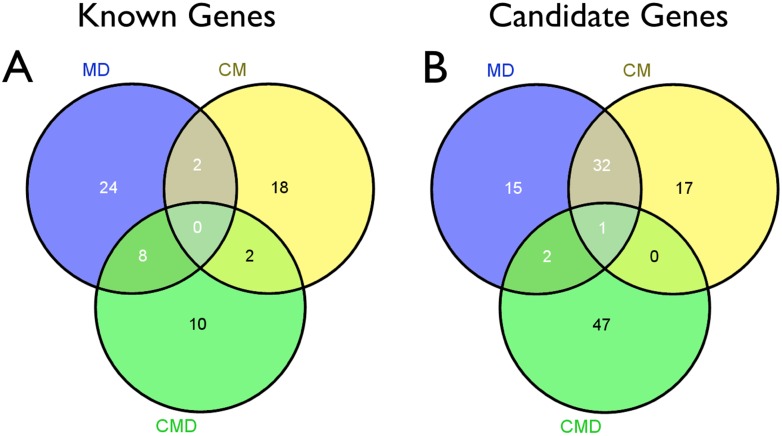
Venn diagrams of gene set overlaps. A: Venn diagram showing the overlap of training set genes between muscular dystrophies (MD), congenital myopathies (CM) and congenital muscular dystrophies (CMD). B: Venn diagram showing the overlap of genes found within the top 50 ranked candidate genes in the three disease groups.

The integrated data mining reveals or confirms allelic diseases. Indeed, while proposed genes for metabolic myopathies or myasthenic syndromes are rather group-specific, a larger overlap occurred between congenital myopathies and muscular dystrophies than what was expected from the analysis of overlap between these groups' training sets. While only 2 genes out of 34 muscular dystrophy training set genes also appeared among the 22 congenital myopathy training set genes (*TTN* and *TRIM32*), the first positions on the ranked lists of candidate genes after filtering for known diseases encompass a large overlap of genes: 5 out of the top 8 candidate genes for muscular dystrophies are within the top 8 for congenital myopathies (Table 4), and 33 out of the top 50 candidate muscular dystrophy genes are also within the top 50 candidate genes for congenital myopathies (Figure 3 and Table S2). Overlaps are also substantial between both these lists and the one for myofibrillar myopathies, but in this case the overlap was expected as the training set for myofibrillar myopathies, despite being small (7 genes), included 2 genes also associated to muscular dystrophies and 1 gene associated to congenital myopathies. On the other hand, while the training sets of muscular dystrophies and congenital muscular dystrophies overlap with a significant share of 8 genes, only 3 genes within the top 50 candidate genes is the same for both groups. The reasons for these results lie in the signature of the disease groups: the breakdown of biological processes terms (depicted in Table 2), which represent the larger share of GO terms, is more comparable between congenital myopathies and muscular dystrophies, with similar contributions of terms involving muscle contraction and development, as opposed to the absence of resemblance between these disease groups and the congenital muscular dystrophies breakdown, which is enriched with mainly non-skeletal muscle-related terms, especially glycosylation. Taken together, gene clustering and candidate genes retrieval suggest that mutations in the same genes will eventually be linked to both muscular dystrophies and congenital myopathies.

Table 4 presents the top 8 ranked genes after excluding genes with known disease annotation. Genes with known disease annotation (listed in Table S2) might still be good candidate genes for myopathies, considering that phenotypic variability is more a rule than an exception for known myopathy genes. This is the case for genes linked to both myopathy and cardiomyopathy. It is thus expected that known cardiomyopathy-associated genes may be found associated with a skeletal muscle phenotype. Such phenotypic variability may even transcend the realm of muscle alteration. *DNM2*, for example, is associated both with centronuclear myopathy, a congenital myopathy, and Charcot-Marie Tooth disease, a hereditary neuropathy [48], [49]. *LMNA*, in addition to multiple myopathic phenotypes, also causes Charcot-Marie Tooth disease or progeria [50], [51], and *SYNE1* can cause one type of Emery-Dreifuss muscular dystrophy, a dilated cardiomyopathy syndrome, a form of autosomal recessive arthrogryposis, and autosomal recessive spinocerebellar ataxia [52]–[55]. The origin of the variability may stem from varying impacts of mutations in different protein domains. For example, in *DNM2*, mutations giving rise to a centronuclear myopathy phenotype are enriched in the interface between the middle domain and the pleckstrin homology domain, while mutations implicated in Charcot-Marie-Tooth disease tend to cluster in other parts of the pleckstrin homology domain [56].

After we carried out this work, a form of vacuolar myopathy was associated to *CLN3*, implicated in neuronal ceroid lipofuscinosis, ranked 343rd for vacuolar myopathies [57]. In addition, *LRP4*, a gene associated with Cenani-Lenz syndactyly syndrome and sclerosteosis, and ranked high (54th) for congenital muscular dystrophies, was implicated in a patient with a CMS disease [58]. We therefore suggest that genes with disease annotation in the ranked lists should be considered with caution in analyzing NGS results, but not *a priori* excluded when using filtering pipelines.

### Example of the usage of the ranked lists

We found the ranked lists to be helpful in our own analysis of exome data to prioritize the scrutiny of potential novel genes implicated in myopathies. The gene ranks in the Excel file sheets in Table S2 can be easily used as additional genomic annotation.

Consider this exome of a sporadic patient affected with nemaline myopathy, a congenital myopathy, from unaffected parents (Table S4). Out of an initial 86,333 variants called in the exome data, 250 remained after variants filtering to exclude purported sequencing errors and polymorphisms. The first variants we analyze closely are the ones found in genes with known implication in myopathies. The heterozygous variants in *DMD* and *CACNA1S* were subsequently found in the unaffected father, while the missense variant in *ANO5*, associated with autosomal recessive limb girdle dystrophy 2L, would require an association to a second mutation to cause disease. We can thus exclude the implication of these known genes.

We next focus on the candidate genes for congenital myopathies (Table 4 and Table S2). If a gene has more than 10% expression in skeletal muscle and is not associated to a disease, it receives a flag as a “candidate”. Only 29 variants, a significant reduction from the original 250, survive this additional filtering and are shown in Table 6.

**Table 6:** Resulting 29 variants after filtration of exome data of a patient affected with nemaline myopathy.

CM Rank	Flag	VariantID	State	Gene	Spec%
208	candidate	12_120660719_C_T	Heterozygous	PXN	22
355	candidate	1_203139425_T_A	Heterozygous	MYBPH	16
446	candidate	4_23803919_C_T	Heterozygous	PPARGC1A	30
586	candidate	5_150028613_T_C	Homozygous	SYNPO	100
610	candidate	5_138160333_G_A	Heterozygous	CTNNA1	34
786	candidate	10_115374035_A_T	Heterozygous	NRAP	100
856	candidate	1_87208087_A_G	Heterozygous	SH3GLB1	100
951	candidate	6_36076169_A_G	Heterozygous	MAPK14	18
1044	candidate	11_1901435_C_T	Heterozygous	LSP1	36
1044	candidate	11_1901435_C_T	Heterozygous	LSP1	36
1199	candidate	9_125863896_C_T	Heterozygous	RABGAP1	38
1758	candidate	12_95604081_G_A	Heterozygous	FGD6	17
1902	candidate	14_103420979_G_A	Heterozygous	CDC42BPB	32
1976	candidate	9_124522285_C_T	Heterozygous	DAB2IP	43
2066	candidate	22_19865895_A_C	Heterozygous	TXNRD2	16
2231	candidate	5_95116054_A_T	Heterozygous	RHOBTB3	32
2245	candidate	22_41652800_A_C	Heterozygous	RANGAP1	25
2263	candidate	2_159477732_C_A	Heterozygous	PKP4	10
2360	candidate	2_152980460_G_T	Heterozygous	STAM2	26
2679	candidate	1_46472006_A_G	Heterozygous	MAST2	100
3075	candidate	10_68138967_C_T	Heterozygous	CTNNA3	19
3434	candidate	7_156976610_G_A	Heterozygous	UBE3C	100
3530	candidate	9_32407367_C_T	Heterozygous	ACO1	16
3627	candidate	1_19453077_C_T	Heterozygous	UBR4	100
4029	candidate	20_35632140_C_G	Heterozygous	RBL1	40
4235	candidate	22_50356432_A_T	Heterozygous	PIM3	47
4375	candidate	11_75115893_C_A	Heterozygous	RPS3	14
5062	candidate	1_204494668_G_A	Heterozygous	MDM4	47
5084	candidate	7_21469915_C_T	Heterozygous	SP4	36

An initial 86,333 variants were reduced to 250 using criteria on the variant level, which resulted in the 29 variants after exclusion of genes already ascribed to diseases and based on specificity of skeletal muscle expression. Variants are then sorted according to the gene ranking calculated for the congenital myopathy group.

The gene with the highest rank was *PXN*, which codes for paxillin, a protein believed to have a function related to integrins and cytoskeletal localization in multiple tissues, skeletal muscle included [59]. The second best gene was *MYBPH*, which codes for myosin-binding protein H, the second most abundant protein of the family of myosin-associated proteins [60]. Except for its cloning, not much is known about its specific function. The third gene, *PPARGC1A*, has regulatory functions on glucose and fat oxidation in muscle cells and protects skeletal muscle fibers against atrophy in mouse models [61]. However, in all these genes, single mutations were found in a heterozygous state, thus a putative dominant negative effect or haploinsufficiency would be required for a pathogenicity call. The next gene in the list, *SYNPO*, produces synaptopodin, a protein whose name stems from its involvement in synapses involving dendritic spines, in addition to renal podocytes [62]. In spite of its name, skeletal muscle is actually the tissue where it is most strongly expressed. Furthermore, synaptopodin directly binds actin, one of the proteins known to be involved in nemaline myopathy. The missense variant found in this gene was homozygous, in a highly evolutionary conserved position, with prediction of pathogenicity in multiple tools, and was Sanger confirmed to be homozygous in the patient and heterozygous in her parents. We believe *SYNPO* is the best candidate gene for this family, based on a recessive scenario. While we cannot discard the other genes, the ranking of candidate genes based on our integrative data mining quickly highlights the best genes to proceed further in functional analysis.

## Conclusions

The above integrated data mining approach was successfully used to retrieve both specific signatures for different myopathy groups and to uncover and rank interesting candidate genes for myopathies. Recent discoveries of gene implications that were correctly identified by the disease group’s signature validated this approach. *In silico* approaches allow for systematic, but modifiable criteria to be used in generating ranked candidate lists and have the added benefit of automation, whereby such lists can be updated on the fly as new knowledge is incorporated in genomic databases.

Signatures and candidate genes highlighted both potential common pathological mechanisms and overlap between several disease groups. In addition, the ranked candidate gene lists are helpful to prioritize functional validation of filtered variants from overwhelming NGS data.

## Supporting Information

Table S1 **Breakdown of descriptor terms for every domain of each disease group, with corresponding calculated weights.**
(XLSX)

Table S2 **Full ranked gene lists for each disease group.**
(XLSX)

Table S3 **Ranked lists of known genes for each disease group.**
(XLSX)

Table S4 **Filtered variants from an exome of a patient with nemaline myopathy, ordered according to the ranked gene lists for congenital myopathies.**
(XLSX)
