# Correction: Integrative Data Mining Highlights Candidate Genes for Monogenic Myopathies

**DOI:** 10.1371/journal.pone.0117884

**Published:** 2015-02-03

**Authors:** The PLOS ONE Staff

There are two errors in this article. The first author’s name is represented incorrectly in the citation and should appear as “Abath Neto O”. The correct citation is: Abath Neto O, Tassy O, Biancalana V, Zanoteli E, Pourquié O, et al. (2014) Integrative Data Mining Highlights Candidate Genes for Monogenic Myopathies. PLoS ONE 9(10): e110888. doi:10.1371/journal.pone.0110888.

There is an error in affiliation 2 for authors Osorio Abath Neto and Edmar Zanoteli. Affilation 2 should be: “Departamento de Neurologia, Faculdade de Medicina da Universidade de São Paulo (FMUSP), São Paulo, Brazil.”
